# Ramadan fasting in Saudi Arabia is associated with altered expression of CLOCK, DUSP and IL-1alpha genes, as well as changes in cardiometabolic risk factors

**DOI:** 10.1371/journal.pone.0174342

**Published:** 2017-04-06

**Authors:** Ghada M. A. Ajabnoor, Suhad Bahijri, Noor Ahmad Shaik, Anwar Borai, Aliaa A. Alamoudi, Jumana Y. Al-Aama, George P. Chrousos

**Affiliations:** 1 Department of Clinical Biochemistry, Faculty of Medicine, King Abdulaziz University, Jeddah, Saudi Arabia; 2 Saudi Diabetes Study Research Group, King Fahd Medical Research Center, King Abdulaziz University, Jeddah, Saudi Arabia; 3 Department of Genetic Medicine, Faculty of Medicine, King Abdulaziz University, Jeddah, Saudi Arabia; 4 Princess Al-Jawhara Al-Brahim Center of Excellence in Research of Hereditary Disorders, King Abdulaziz University, Jeddah, Saudi Arabia; 5 King Abdullah International Medical Research Center (KAIMRC), College of Medicine, King Saud Bin Abdulaziz University for Health Sciences (KSAU-HS), Jeddah, Saudi Arabia; 6 First Department of Pediatrics, University of Athens Medical School,‘‘Aghia Sophia” Children’s Hospital, Athens, Greece; University of Lübeck, GERMANY

## Abstract

**Background:**

During the fasting month of Ramadan, practicing Saudis develop severe disturbances in sleeping and feeding patterns. Concomitantly, cortisol circadian rhythm is abolished, diurnal cortisol levels are elevated and circulating levels of several adipokines are altered favouring insulin resistance.

**Aim:**

To examine changes in the expression of CLOCK and glucocorticoid-controlled genes, such as DUSP1 and IL-1α in Saudi adults before and during Ramadan, and to investigate possible associations with selected cardiometabolic risk factors.

**Methods:**

Healthy young volunteers (5 females, 18 males; mean age +SEM = 23.2 +1.2 years) were evaluated before Ramadan and two weeks into it. Blood samples were collected at 9 am (±1 hour) and twelve hours later for determination of serum lipid profile, high sensitivity CRP (hsCRP), and adiponectin. The expression of CLOCK, DUSP1 and IL-1α was evaluated in circulating leukocytes.

**Results:**

Mean levels of GGT and morning adiponectin decreased, while those of LDL-c/ HDL-c and atherogenic index (AI) increased significantly in Ramadan compared to Shabaan. There was no significant difference between morning and evening adiponectin during Ramadan, while the diurnal rhythm of hsCRP was lost. CLOCK gene expression mean was significantly higher in morning than in evening during Shabaan. Mean morning and evening DUSP1 mRNA levels showed significant increase during Ramadan compared to Shabaan, however, its diurnal rhythm was maintained. Morning IL-1α mRNA expression remained significantly higher than in the evening during Ramadan, but was markedly decreased compared to Shabaan.

**Discussion:**

Ramadan fasting in Saudi Arabia is associated with improvements in some cardiometabolic risk factors, such as circulating GGT and hsCRP and leukocyte expression of IL-1α mRNA, suggesting that intermittent fasting might have a beneficial component. These benefits may be offset by the previously reported dysregulation in the circadian rhythm, excess glucocorticoid levels and action, and insulin resistance, explaining increased prevalence of cardiometabolic disorders and type 2 diabetes mellitus.

## Introduction

Circadian rhythms control many physiologic processes, including energy metabolism, hormone biosynthesis, and immune responses. It is estimated that approximately 10% of the main transcriptome in mammalian cells is expressed with a circadian rhythm. Indeed, studies examining the physiologic effects of sleep restriction, reported changes in the circadian timing system [1], as well as immune [2] and endocrine [3, 4] variables.

In the recent years, the molecular mechanisms that control the circadian rhythm, also known as the “molecular clock”, have been identified [5]. These consist of a series of interlocked transcriptional-translational feedback loops, with one of the major loops being the CLOCK/BMAL1 loop [6, 7]. Circadian clock genes are expressed in a circadian fashion in the suprachiasmatic nucleus (SCN) of mammals, constituting the “master” circadian pacemaker of the organism. The important link between the CLOCK gene and intermediary metabolism has been shown in CLOCK knock-out mice, which have impaired glucose tolerance and insulin secretion, resulting in a diabetic phenotype [8].

Current work suggest a major role for shift-work induced alterations in core circadian clock genes [9, 10] in disrupting circadian metabolic regulation, and in inducing various shift-work associated diseases [11–13]. In a similar manner to shift workers, during the fasting month of Ramadan, Muslims in Saudi Arabia experience severe disturbance in their sleeping patterns, with loss of night-time sleep and shortening of sleep duration. This can be associated with the loss of the circadian rhythm of cortisol, a hormone that controls the expression of many other hormones, and cytokines including adipokines [14]. This loss of circadian rhythmicity might be related to alterations in the expression of clock genes, resulting in hypercortisolism and chronic smoldering inflammation, increasing the risk of chronic cardiometabolic disorders.

One of many important genes upregulated by cortisol is the Dual Specific Phosphatases (DUSP) gene [15]. DUSP comprise a group of enzymes that can dephosphorylate both tyrosine and serine/threonine residues of their protein substrates. A big subset of the DUSP family contain in addition to the common phosphatase domain a mitogen activated protein kinases (MAPK) binding domain, allowing it therefore to dephosphorylate, and thus deactivate MAPK [16]. MAPK proteins are protein kinases that respond to various cell stimuli such as; mitogenic signals, pro-inflammatory cytokines, and oxidative/heat stress to regulate various cell functions. Thus, through regulating MAPK, DUSP proteins play an important role in cell responses to environmental stress. Indeed, DUSP can through negative regulation prevent the deleterious effect of a persistent innate immune response [17].

IL-1 is one of the most potent innate immunity cytokines, playing a key role in inflammation initiation and perpetuation [18]. This cytokine is predominantly secreted by innate immune cells and acts to activate both innate and adaptive immune responses.It has also been associated with autoimmunity and auto-inflammation, including type 2 diabetes mellitus. The importance of the circadian clock in IL-1β expression has been demonstrated in Per2 deficient mice, which showed higher levels of IL-1β and IFN-γ in the serum, and a stronger vulnerability to LPS-induced endotoxin shock than wild type mice [19, 20].

Caloric restriction (CR) and intermittent fasting (IF) have been reported to improve several risk factors for stroke and coronary artery diseases, including a reduction in blood pressure and increased insulin sensitivity [9, 10, 21]. However, taking into account the sleep restriction and nocturnal eating during Ramadan in Saudi Arabia, it is imperative to understand the effects of circadian rhythm alterations on various physiologic systems, and to estimate their possible contribution to the overall health and quality of life outcomes of Saudi Ramadan practitioners.

The effects of disrupted sleep and feeding patterns during Ramadan on the expression of CLOCK, and glucocorticoid-controlled genes, such as DUSP1 and IL-1 have not been studied before. Therefore, we examined the changes in the diurnal expression of these three genes in healthy young Saudi adults before and two weeks into Ramadan. In addition, we investigated possible associations with selected cardiometabolic risk factors, both novel, such as Gamma glutamyl transferase (GGT) and adiponectin, and traditional ones, such as high sensitivity C-reactive protein (hsCRP), atherogenic index (AI), and the LDL-c:HDL-c ratio.

## Subjects and methods

### Study design

Full description of study design, as well as physiologic and anthropometric characteristics of the studied subjects were outlined in detail in our earlier report [14]. In summary, subjects were studied twice; before fasting during their regular life (Shaaban), and again 10–15 days after starting the fast (Ramadan). Care was taken to ensure that the participating females were in the same menstrual phase during both visits. The study was approved by the "Committee on the Ethics of Human Research" at the Faculty of Medicine- King Abdulaziz University, and a written informed consent was signed by all participants. Reference ranges for intended measurements, and laboratory quality control data were used to calculate sample size to avoid type II statistical error [22]. Twenty-three of the originally recruited healthy volunteer subjects (18 males, 5 females), aged 18–42 years completed the study. To avoid group variability at the study baseline the same subjects were studied at different times which also helped in decreasing the number of subjects needed. Subjects were instructed to have meals as usual on the day of testing, and to record their usual sleeping and waking times of the previous three days, thus helping to control for the effects of diet and sleep patterns on measured parameters.

Blood sampling was performed twice daily, at 9 am ± 1 hour and again twelve hours later. Therefore, the first and third samples were during fasting state (at least 10 hours for sample one and 6–7 hours for sample 3), while the second and fourth samples were 2–5 hours after meals.

Blood samples were drawn into plain tubes and serum was separated for measurement of total and HDL cholesterol, triglycerides, hsCRP, GGT and adiponectin. In addition, 2.5 ml of blood were drawn in vacutainer heparin tubes containing 2 ml Trizol reagent from 10 subjects to estimate diurnal mRNA expression of CLOCK, DUSP 1, and IL-1. Samples were stored at -80°C until measurements were performed.

### Biochemical and endocrine assays

All serum biochemical parameters were assayed in the accredited Clinical Chemistry Laboratory at the National Guard Hospital-King Abdulaziz Medical City-Jeddah, KSA. GGT and lipids (cholesterol, triglycerides and HDL-C were assayed in collected serum samples using an ABBOTT Architect c16000 auto-analyzer. The same auto-analyzer was utilized to measure hsCRP, employing immunoturbidimetric determination technique. LDL-C was calculated using the Friedewald equation [23]. The LDL\ HDL ratio was calculated by dividing each estimated value of LDL-C by the corresponding calculated HDL-C value, while the AI was estimated by using the equation AI = log (TG/HDL-C) [24].

The concentrations of adiponectin in serum samples were assayed at the “Nutrition Research Unit” laboratory at King Fahd Medical Research Center. Serum adiponectin was measured using the “Biovendor human adiponectin ELISA” high sensitivity kit. The assay was a solid phase enzyme-linked immunosorbent assay, and was carried out according to the manufacturers’ procedure. Absorbance was measured at 450 ± 10 nm using a microplate reader (Biokit®, ELX800- USA).

### Total RNA isolation and SYBR Green real-time PCR

To determine mRNA levels of CLOCK, DUSP1, and IL 1 in peripheral blood mononuclear cells (PBMCs), quantitative real-time reverse transcriptase-polymerase chain reaction (RT-PCR) was performed using the ABI PRISM 7000 according to the manufacturer’s standard protocol (Applied Biosystems) at Princess Al-Jawhara Al-Brahim Centre of Excellence in Research of Hereditary Disorders (PACER-HD). Total RNA was purified from PBMCs using Trizol Reagent (Promega), according to the manufacturer’s instructions. Quantitative PCR analysis was carried out using SYBR® Green PCR Master Mix (One-step, Life Technologies Corporation Carlsbad, CA). Primers were designed and obtained from BioServe Biotechnologies (India) Pvt Ltd. All primer pairs used for measuring mRNA levels of CLOCK, and glucocorticoid-responsive genes, as well as the primer pairs for beta-actin, glyceraldehyde-3-phosphate dehydrogenase (GAPDH) and ribosomal protein large P0 (RPLP0) mRNAs are shown in Table 1. The obtained Ct (threshold cycle) values of these mRNAs were normalized for mean Ct values of the beta-actin, GAPDH and RPLPO mRNAs, and their relative expressions were shown as fold induction or change over the mean values of all subjects.

**Table 1:** Primers pairs used in real-time mRNA quantitation.

Gene		Primer sequence
CLOCK	Forward	5`-GAA GTT AGG GCT GAA AGA C-3`
	Reverse	5`-GAT CAA ACC TTT CCA ATG C-3`
IL-1	Forward	5`-GAC CTG AAG AAC TGT TAC AG-3`
	Reverse	5`-GAT CCA TGC AGC CTT CAT G-3`
DUSP1	Forward	5`-CAA GTC TTC TTC CTC AAA GG-3`
	Reverse	5`-GAA CTG CAC CCA GAT TCC-3`
BETA-ACTIN	Forward	5`-CAA CCG CGA GAA GAT GAC-3`
	Reverse	5`-GTC ACC GGA GTC CAT CAC-3`
GAPDH	Forward	5`-CAA TGA CCC CTT CAT TGA C-3`
	Reverse	5`-GAT GGT GAT GGG ATT TCC-3`
RPLO	Forward	5`-CCA GCT CTG GAG AAA CTG-3`
	Reverse	5`-CTT CAC ATG GGG CAA TGG-3`

Primers obtained from BioServe Biotechnologies (India) Pvt Ltd

### Statistical analyses

Analyses were performed using SPSS statistical package version 19. Descriptive statistics, such as mean ± SEM, were calculated for all estimated parameters. Paired Student t-test was employed for comparison of means between months, days and nights. Significance was assigned at p<0.05.

## Results

Results of estimated and calculated biochemical parameters estimated at different points of the study are presented in Table 2 (S1 Dataset).

**Table 2:** Estimated and calculated biochemical parameters estimated at different points of the study.

		Shaaban		Ramadan		
		Mean ± SEM	P value (AM-PM)	Mean ± SEM	P value (AM-PM)	P value Shaaban-Ramadan
Adiponectin* (μmol/ml)	AM	11.62±0.80	**0.001**	8.80±0.57	0.375	**0.001**
	PM	8.61±0.67		9.28±0.78		0.405
**hsCRP⃰ (mg/L)**	AM	1.68±0.47	**0.008**	0.97±0.22	0.492	0.089
	PM	1.33±0.32		1.13±0.35		0.476
**GGT⃰ (U/L)**	AM	27.6±4.3		22.9±3.9		**0.018**
**LDL-c/ HDL-c**	AM	2.50 ± 0.125		2.78 ± 0.125		**0.01**
**Atherogenic index (AI)**	AM	-0.204 ±0.047		0.010 ±0.044		**0.01**

GGT, gamma glutamyl transferase; hsCRP, high sensitivity C-reactive protein; (*)

There was a significant decrease in GGT and morning adiponectin in Ramadan compared to Shabaan reported earlier in Ajabnoor et al. 2014 [25].

The means of the LDL-c/ HDL-c ratio and AI increased significantly in Ramadan compared to Shabaan. In addition, there was no significant difference between morning and evening levels of adiponectin during Ramadan reported earlier in Ajabnoor et al. 2014 [25].

There was also a loss of diurnal rhythm of hs-CRP, with lower values being noted during Ramadan, but no significant difference in mean values compared to Shabaan reported earlier in Ajabnoor et al. 2014 [25].

**Fig 1:**
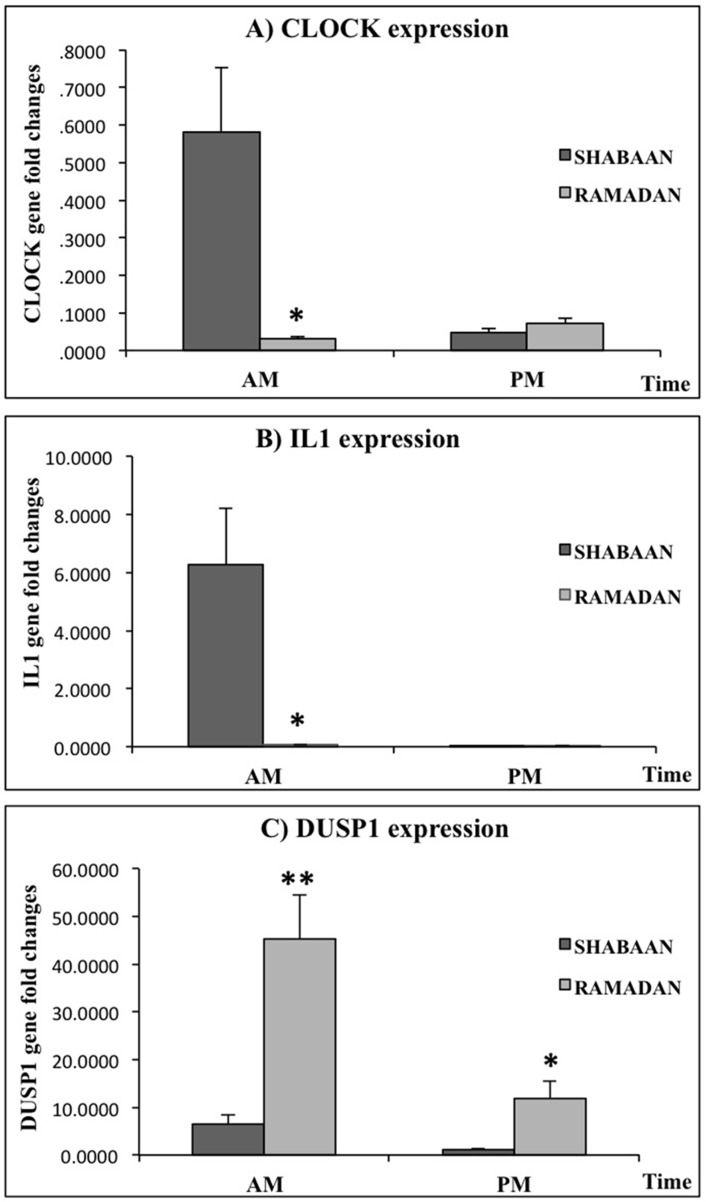
The analysis of CLOCK, IL1 and DUSP gene expression in the morning and evening during the months of Ramadan and Shabaan. The expression levels of A)CLOCK, B)IL-1, and C)DUSP genes in the PBMCs of healthy volunteers (N = 23) in the morning (AM) and evening (PM) during Ramadan and Shabaan. Expression levels were normalized to housekeeping genes (β-actin and GADPH), and expressed as fold changes over the mean values of all subjects. Statistical analysis was performed using Student T test, values shown are mean ±SEM, *P<0.05 or **P<0.*01*.

The expression of the three selected genes during Shabaan and Ramadan is presented in Fig 1, and Table 3 (S2 Dataset).

**Table 3:** The expression of CLOCK, IL -1, and DUSP 1 genes in the morning and evening during Shabaan and Ramadan expressed as mean ± SEM.

		Shaaban		Ramadan		Shaaban-Ramadan
		Mean ± SEM	P value (AM-PM)	Mean ± SEM	P value (AM-PM)	P value
CLOCK	AM	0.5811±0.17190	**0.015**	0.0306 ±0.00702	**0.041**	**0.010**
	PM	0.0476 ±0.01188		0.0720 ±0.01347		0.248
IL-1	AM	6.2825 ±1.91581	**0.011**	0.0706 ±0.01725	**0.008**	**0.012**
	PM	0.0110 ±0.00349		0.0173 ±0.00405		0.389
DUSP1	AM	6.4428 ±2.05352	**0.034**	45.3719 ±9.17392	**0.008**	**0.002**
	PM	1.1254 ±0.23723		11.7685 ±3.63662		**0.017**

The mean of CLOCK was significantly higher in the morning during Shabaan, but this was reversed in Ramadan. Furthermore, morning, but not evening mean in Shabaan was significantly higher than the corresponding mean during Ramadan. On the other hand, mean morning and evening DUSP 1 showed a significant increase during Ramadan compared to their corresponding means during Shabaan; however, the diurnal rhythm was maintained with morning means remaining significantly higher during both months. Similarly, the mean morning IL-1 remained significantly higher than evening mean during Ramadan. However, the morning mean during Ramadan was markedly decreased compared to Shabaan.

## Discussion

It is believed that shift work uncouples the biological circadian clock and the external world zeitgebers, which, in turn, may contribute to shift-work associated diseases [11–13].

Similar to shift workers, Saudi adults practicing fasting during the month of Ramadan experience severe disturbance in their sleeping patterns. In our study we found profound changes in the diurnal expression of CLOCK,a central componenet of the circadian molecular clock, during Ramadan compared to the non-fasting month of Shabaan(Table 3, Fig 1A). In addition to the expected reversal of pattern, with a higher mean in the evening during Ramadan compared to a higher morning mean in Shabaan, a significant decrease in morning mean during Ramadan was noted compared to Shabaan. This is consistent with recent studies observing daily oscillation expression in peripheral tissues of some circadian CLOCK gens through transcriptional-translational feedback loops to control circadian rhythms [26]. Indeed, several studies have also supported a significant cyclic rhythm of clock genes expression such as PER1, PER2,PER3, and BMAL1 in PBMCs of healthy subjects. This diurnal expression was characterized by a peak expression of the PER genes during the usual time of activity in the morning [26]. However, only a few of those studies have commented on CLOCK gene expression. In a study by Takata et al, human CLOCK gene expression in PBMCs from health volunteers exhibited no daily variations when compared to PER2 gene expression [27]. Moreover, it is worth noting that one limitation of the majority of these studies is the limited number of subjects examined.

In a mouse model that lacks a functional CLOCK, the loss of circadian function was associated with impaired glucose tolerance and insulin secretion resulting in a diabetic phenotype [28]. Thus, our previously reported changes in insulin secretion patterns and increase in insulin resistance during Ramadan [14, 25], could be explained by the noted decrease in the morning and evening expression of CLOCK found in this study (Table 3, Fig 1A).

The peripheral CLOCK- mediated circadian acetylation of human glucocorticoid receptor (GR) act as target-tissue and their mechanism of action is diurnally fluctuating by cortisol which is high in morning and low at night [11, 12].

Evening elevations of cortisol as reported in Ramadan [14], and similar to that reported due to night-shift work are associated with uncoupling of the SCN CLOCK- directed HPA axis activity from the daily oscillation of target tissue sensitivity to glucocorticoids, producing functional hypercortisolism and, hence, multiple components of the metabolic syndrome with resultant cardiovascular complications [12, 13, 22]. We have already reported deleterious changes in components of the metabolic syndrome during Ramadan, namely increases in triglycerides and decreases in HDL-c [14]. In this study, we calculated both LDL-c/ HDL-c ratio and AI; both cardiometabolic risk indicators reported to carry greater predictive value than isolated parameters used independently, particularly LDL [24, 29, 30]. Other studies in populations differing in ethnicity have confirmed their value [31, 32]. Therefore, our noted increase in the means of LDL-c/ HDL-c ratio and AI, during Ramadan further confirms the increased cardiometabolic risk compared to pre-fasting month.

Glucocorticoids were reported to upregulate the expression of DUSP1 gene [33], which could explain the upregulation of DUSP expression in the morning and evening during Ramadan in our study. DUSP deactivates MAPKs [16], downregulating the innate immune response [17]. Therefore, the changes in DUSP expression could explain the downregulation in the expression of IL-1, which acts to activate both the innate and adaptive immune response [18]. Furthermore, it could explain our earlier reported lower mean IgG during Ramadan [34]. This is of particular importance to diabetics and the elderly possibly rendering them more susceptible to infectious diseases.

GGT is a common biomarker of liver injury and liver diseases. However, studies have also indicated that GGT might be an early marker of oxidative or other cellular stress, hence linking it to the pathogenesis of type 2 diabetes mellitus and arterial hypertension; perhaps as an oxidative stressor itself. More recent studies reported a strong association between GGT and hypertension and associated heart diseases [19]. Indeed, several studies and meta-analyses have showed positive correlations between GGT levels and the risk of cardiovascular disease, diabetes mellitus and metabolic syndrome [19] [35]. In our study there was a noted decrease in the mean GGT during Ramadan, which could be explained by the effects of fasting [36], changes in dietary pattern [37] on protein synthesis in the liver, and/or changes in the pattern of cortisol secretion [33]. Whatever the cause, the lower mean during Ramadan might validate earlier reports of the cardiometabolic benefits of intermittent fasting [9, 10, 21].

CRP is a highly sensitive inflammatory marker that is produced mainly by the liver in response to infection, inflammation and trauma. CRP is one of the acute phase proteins that plays an important role in host defense by binding to pathogens and damaged tissue, thus aiding their clearance [38, 39]. CRP can activate the complement system and bind to FC receptors leading to the generation of pro-inflammatory responses. Morning hsCRP levels are reported to be higher than those measured at midday or evening [28]. Circulating hsCRP levels are low in healthy individuals; however, levels can rapidly rise in response to inflammation, and similarly can rapidly decline following the resolution of the condition, making it an ideal marker to monitor various inflammatory conditions. In particular, hsCRP has been considered a marker of cardiovascular disorders, and has been linked to the pathophysiology of atherosclerotic plaque formation. Disturbances in sleeping patterns were reported to increase CRP [40]. However, we found generally lower hsCRP values in Ramadan, accompanied by loss of the evening decrease noted in Shabaan. Such changes are beneficial and decrease cardiovascular risk [41]. Since the expression of CRP is mainly induced by IL-6, and enhanced by IL-1β [39], the noted decrease could be linked to decreased expression of IL-1 during Ramadan. Nevertheless, the earlier reported changes in cortisol secretion [14] could also play a role [33].

The circulating levels of the adipokine adiponectin have been reported to decrease insulin resistance [42], with its secretion by cultured adipocytes inhibited by pro-inflammatory cytokines [43]. Adiponectin has also been reported to be anti-atherogenic by: 1) regulating the main signaling pathways involved in the genesis of atherosclerosis [44], 2) suppressing monocyte adhesion to the vascular endothelium [45], and 3) promoting angiogenesis in endothelial cells [46]. In addition, it has been shown to have anti-inflammatory properties which may regulate steps in the atherogenic process [46]. Moreover, adiponectin has been reported to inhibit macrophage conversion to foam cells, and to reduce oxidation of low density lipoprotein (LDL) [47]. It has also been reported that decreased levels of adiponectin are associated with hypertension through various mechanisms [48]. Therefore, the dysregulation in adiponectin production may be an important factor in endothelial dysfunction, increasing the risk of CVD. Indeed, human studies have found that increased plasma adiponectin correlates with a reduced risk of myocardial infarction in men [49], and a lower risk of coronary heart disease in diabetics [50]. In our study, we noted a decrease in the mean morning level of adiponectin, with no significant difference between morning and evening level during Ramadan (Table 2). These changes were associated with increased insulin resistance [25], and could increase risk of CVD. Unfortunately, we did not measure the possible effects on blood pressure or circulating adhesion molecules, however the increased LDL-c/HDL-c ratio and AI support our suggestion.

In conclusion, Ramadan fasting in Saudi Arabia is associated with improvements in certain cardiometabolic risk factors, such as GGT, hsCRP and expression of IL 1, suggesting that intermittent fasting might be more beneficial than increased small meal frequency as suggested by some. However, it seems that the possibly intended benefits of intermittent fasting, as directed by religious teachings, may be offset by the dysregulation in the diurnal rhythm during Ramadan. This might partially explain the phenomenal increase in the prevalence of hypertension, dyslipidemia, metabolic syndrome and type 2 diabetes mellitus in Saudi Arabia. Therefore, to maximize health benefits of IF, it is highly recommended to control the present day sleep disturbance during Ramadan. Firmer conclusions could be made if a similar study is carried out in a country in which sleep patterns are not affected during Ramadan.

## Supporting information

S1 Dataset Biochemical data. (PDF)

S2 Dataset QPCR data. (PDF)
