# Supplementary material for: Ramadan fasting in Saudi Arabia is associated with altered expression of CLOCK, DUSP and IL-1alpha genes, as well as changes in cardiometabolic risk factors
Source: PLoS One. 2017 Apr 6;12(4):e0174342. doi: 10.1371/journal.pone.0174342 (PMC5401765; doi:10.1371/journal.pone.0174342)
Supplement: S1 Dataset — (PDF) [file pone.0174342.s001.pdf]

S1 Biochemical Data

S_N	S_UHDL_AM	S_Chol_AM	S_Trig_AM	LDL_S_AM	S-LDL/HDL -AM	S-AI-AM	R_UHDL_AM	R_Chol_AM	R_Trig_AM	LDL_R_AM	R-LDL/HDL	R-AI-AM	S-GGT-AM	S-CRPhs-AM	S-CRPhs-PM	R-GGT-AM	R-CRPhs-AM	R-CRPhs-PM	S-Adipo-AM	S-Adipo-PM	R-Adipo-AM	R-Adipo-PM
S01	1.39	4.49	0.62	2.82	2.03	-0.35	1.19	4.14	0.57	2.76	2.32	-0.32	27	0.4	0.28	22	0.77	0.76	10.509	7.551	9.885	9.816
S02	1.13	4.49	0.61	3.08	2.73	-0.27	0.92	4.81	2.84	3.59	3.90	0.49	23	1.12	1.0	17	1.95	1.91	9.84	6.15	6.408	6.942
S03	1.17	4.50	0.89	2.92	2.50	-0.12	1.12	4.82	0.85	3.42	3.05	-0.12	11			10	0.4	0.34	15.747	12.69	10.152	11.22
S04	1.29	4.46	0.71	2.85	2.21	-0.26	1.13	4.35	1.05	2.62	2.32	-0.03	31	0.28	0.21	25	0.57	0.39	2.958	14.556	9.282	7.677
S05	0.83	3.54	0.43	2.51	3.03	-0.29	0.87	3.57	0.99	2.25	2.59	0.06	63	2.31	1.88	11	0.83	0.65	16.581	12.144	8.013	3.669
S06	1.33	4.49	0.76	2.81	2.12	-0.24	1.36	4.75	1.25	2.60	1.91	-0.04	28	4.02	3.61	23	2.04	5.24	10.665	8.409	8.682	8.547
S07	1.10	6.59	0.52	5.25	4.78	-0.33	1.07	6.35	1.30	5.14	4.80	0.08	28	2.7	1.46	31	1.82	1.09	10.041	6.228	9.348	5.406
S08	0.94	4.42	2.72	2.24	2.38	0.46	1.04	5.23	1.80	3.08	2.96	0.24	27			24	0.1	0.13	8.952	7.161	3.801	7.479
S09	1.06	4.16	0.61	2.82	2.66	-0.24	0.98	4.18	1.21	2.79	2.84	0.09	27	9.55	6.12	26	2.28	4.28	14.325	6.852	9.348	9.348
S10	1.42	4.23	0.88	2.41	1.70	-0.21	1.17	4.74	1.06	2.87	2.45	-0.04	20	0.43	0.6	16	0.52	0.49	12.222	6.618	10.086	10.752
S11	1.17	3.57	0.57	2.14	1.83	-0.31	1.10	3.40	0.66	1.83	1.66	-0.22	32	0.57	0.6	29	0.53	0.4	14.169	12.144	12.291	16.434
S12	1.14	6.09	1.08	4.46	3.91	-0.02	0.85	4.48	1.48	3.40	4.00	0.24	19	0.46	0.49	13	0.16	0.24	14.4	6.54	3.669	8.346
S13	1.07	3.56	0.60	2.22	2.07	-0.25	1.04	3.84	0.77	4.08	3.92	-0.13	18	1.4	0.87	18	1.29	0.99	16.176	15.957	10.887	14.094
S14	1.71	6.17	1.45	3.80	2.22	-0.07	1.44	5.63	1.50	3.56	2.47	0.02	17	0.64	0.4	15	0.41	0.41	7.068	4.593	5.538	5.94
S15	1.34	3.87	0.53	2.29	1.71	-0.40	1.17	3.86	0.82	2.39	2.04	-0.15	13	0.97	0.82	12	0.33	0.27	10.431	10.026	8.883	11.154
S16	1.21	4.56	0.90	2.94	2.43	-0.13	1.00	4.26	1.32	2.84	2.84	0.12	26	0.61	0.58	11	0.59	0.43	14.868	9.459	11.355	9.549
S17	0.99	4.29	0.71	2.98	3.01	-0.14	1.01	4.74	1.34	2.69	2.67	0.12	21	1.4	1.19	20	2.06	2.22	10.353	5.451	8.415	9.282
S18	1.38	5.54	0.70	3.84	2.78	-0.29	1.37	5.62	0.55	4.04	2.95	-0.40	20	0.3	0.23	17			19.539	11.055	12.891	17.238
S19	0.79	3.03	0.60	1.97	2.49	-0.12	0.86	3.73	1.33	2.55	2.96	0.19	17	2.16	2.51	14	0.46	0.53	9.42	5.295	9.75	11.622
S21	1.14	5.53	2.19	3.39	2.97	0.28	1.05	5.12	2.09	3.11	2.96	0.30	94	3.83	3.5	92	3.13	2.91	5.769	5.046	3.936	0.795
S22	1.09	3.97	0.60	2.61	2.39	-0.26	1.03	3.60	1.19	2.37	2.30	0.06	12	0.43	0.36	10	0.16	0.21	11.754	7.356	8.883	9.216
S23	1.45	4.55	0.38	2.93	2.02	-0.58	1.20	4.11	0.58	2.83	2.36	-0.32	11	1.71	1.02	9	0.71	0.6	13.389	10.353	13.494	11.556
S24	1.54	3.85	0.43	2.11	1.37	-0.55	1.35	4.42	1.30	2.29	1.70	-0.02	51	0.17	0.2	62	0.4	0.33	8.097	6.306	7.41	7.344
