# Supplementary material for: Ramadan fasting in Saudi Arabia is associated with altered expression of CLOCK, DUSP and IL-1alpha genes, as well as changes in cardiometabolic risk factors
Source: PLoS One. 2017 Apr 6;12(4):e0174342. doi: 10.1371/journal.pone.0174342 (PMC5401765; doi:10.1371/journal.pone.0174342)
Supplement: S2 Dataset — (PDF) [file pone.0174342.s002.pdf]

S2 QPCR Data

	SHAM_IL_delta_ct	SHPM_IL_delta_ct	RMAM_IL1_delta_ct	RMPM_IL1_delta_ct	SHAM_CLOCK_delta_ct	SHPM_CLOCK_delta_ct	RMAM_CLOCK_delta_ct	RMPM_CLOCK_delta_ct	SHAM_DUSP_delta_ct	SHPM_DUSP_delta_ct
S01	1.5	0.03621401	0.024568346	0.000616182	1.25845497	0.006822643	0.07206909	0.033343509	2.005879954	2.08
S02	1.520057582	0.004059386	0.110582024	0.007574228	0.619857718	0.057548051	0.037832657	0.04521256	17.58977381	1.654376243
S03	10.71807636	0.002501875	0.002598553	0.014187972	1.121691634	0.021345745	0.007892114	0.177499872	9.163144326	0.804615627
S04	0.958294778	0.011002656	0.015552825	0.029974161	0.145641106	0.116831723	0.02223676	0.039154894	2.620723332	1.814920903
S05	13.37531399	0.001860213	0.001772954	0.007213468	0.105216884	0.01386901	0.008328882	0.036406041	2.68	0.836915189
S06	10.5368698	0.010422822	0.09675473	0.012837117	0.054927014	0.084182192	0.036247553	0.077783452	1.761076705	0.285592869
S07	12.60143542	0.009235193	0.077949502	0.040677575	0.7177119	0.039246523	0.060992798	0.083010254	0.104595124	0.943380064
S08	0.43602834	0.011750588	0.125193006	0.030456309	0.038079092	0.017166643	0.010114669	0.093168304	7.920920966	1.6647338
S09	2.99646898	0.004175059	0.147089289	0.0386418	0.271485316	0.088632914	0.016449704	0.068389519	14.13921851	0.043671785
S10	1.32	0.03681401	0.103696849	0.023279328	1.4237982	0.03	0.03345633	0.065977126	1.32184551	0.033343509
S11	1.54	0.04059386	0.156789	0.002598553	1.25275497	0.02134021	0.006822643	0.116831723	4.892917846	0.04521256
S12	1.68	0.039015	0.16888312	0.015552825	0.3247553	0.1175467	0.027548051	0.01386901	16.353875	0.177499872
S13	14.88	0.003200266	0.1800865	0.001772954	0.60992798	0.01386901	0.021345745	0.184182192	15.2613708	0.285592869
S14	1.3	0.003826021	0.1	0.009675473	0.64114669	0.078316218	0.030456309	0.039246523	15.4421021	0.943380064
S15	14.2	0.010422822	0.007853302	0.027949502	0.316316884	0.039246523	0.0396418	0.17166643	1.376012	1.6647338
S16	3.2	0.003523519	0.03658	0.012519301	0.054927014	0.006822643	0.023279328	0.098632914	1.642376243	0.043671785
S17	10.89	0.003975088	0.02	0.014452211	0.7177119	0.057548051	0.009892114	0.03	12.84615627	1.285592869
S18	11.73	0.00123438	0.1	0.020324685	0.028079092	0.021345745	0.04323676	0.037548051	13.5149209	1.543380064
S19	12.22	0.001106234	0.01776321	0.017322	0.271485316	0.077783452	0.008328882	0.021345745	2.636915189	1.647338
S21	15.4567889	0.004218001	0.00003589	0.0175088	1.5087982	0.083010254	0.046247553	0.042783452	1.285592869	0.043671785
S22	0.32527897	0.00085032	0.015552825	0.00013438	0.607853218	0.073168304	0.074892698	0.121010254	1.9380064	2.438087982
S23	0.8789896	0.00435193	0.01772954	0.01106234	1.123491634	0.007892114	0.013214669	0.0521304	1.67338	3.24775497
S24	0.23456878	0.009335193	0.09675473	0.0421001	0.1541106	0.02223676	0.05427014	0.00782114	0.013671785	2.35547553

RMAM_DUSP_delta_ct	RMPM_DUSP_delta_ct
1.038443252	23.46314167
73.02116431	10.97801591
50.90377907	25.98283113
70.26774779	16.70241855
66.82442174	23.76607879
1.212381275	2.369884551
60.85088072	0.892917846
44.13105186	1.499593875
40.0972928	0.261370801
1.0443252	6.369884551
71.0211631	0.982917846
50.05779071	1.99593875
71.76779	0.361370801
66.2542174	2.499593875
1.212381275	0.34370801
73.2116431	20.283113
52.3779071	21.70341855
61.26774779	27.07607879
72.2342174	4.369884551
1.056	10.7801591
54.11310519	27.28389
1.456789963	15.241855
58.13105186	25.4668979
